# Comprehensive Analysis and Experimental Validation of a Novel Estrogen/Progesterone-Related Prognostic Signature for Endometrial Cancer

**DOI:** 10.3390/jpm12060914

**Published:** 2022-05-31

**Authors:** Jing Yu, Hong-Wen Yao, Ting-Ting Liu, Di Wang, Jian-Hong Shi, Guang-Wen Yuan, Sai Ma, Ling-Ying Wu

**Affiliations:** 1 Department of Gynecologic Oncology, National Cancer Center/National Clinical Research Center for Cancer/Cancer Hospital, Chinese Academy of Medical Sciences and Peking Union Medical College, Beijing 100021, China; 17611768237@126.com (J.Y.); absurgeonyao@163.com (H.-W.Y.); william327@126.com (G.-W.Y.); 2 Department of Blood Grouping, Beijing Red Cross Blood Center, Beijing 100088, China; liuting2018@yeah.net; 3 The Fourth Affiliated Hospital, Zhejiang University School of Medicine, Yiwu 322000, China; 15604011202@163.com; 4 State Key Laboratory of Molecular Oncology, National Cancer Center/National Clinical Research Center for Cancer/Cancer Hospital, Chinese Academy of Medical Sciences and Peking Union Medical College, Beijing 100021, China; jianhong_sjh@163.com; 5 Department of Laboratory, The Affiliated Suzhou Hospital of Nanjing Medical University, Suzhou 215000, China; 6 Gusu School, Nanjing Medical University, Suzhou 215000, China

**Keywords:** EC, estrogen, progesterone, signature, TCGA

## Abstract

Estrogen and progesterone are the major determinants of the occurrence and development of endometrial cancer (EC), which is one of the most common gynecological cancers worldwide. Our purpose was to develop a novel estrogen/progesterone-related gene signature to better predict the prognosis of EC and help discover effective therapeutic strategies. We downloaded the clinical and RNA-seq data of 397 EC patients from The Cancer Genome Atlas (TCGA) database. The “limma” R package was used to screen for estrogen/progesterone-related differentially expressed genes (DEGs) between EC and normal tissues. Univariate and multivariate Cox proportional hazards regression analyses were applied to identify these DEGs that were associated with prognosis; then, a novel estrogen/progesterone-related prognostic signature comprising *CDC25B*, *GNG3*, *ITIH3*, *PRXL2A* and *SDHB* was established. The Kaplan–Meier (KM) survival analysis showed that the low-risk group identified by this signature had significantly longer overall survival (OS) than the high-risk group; the receiver operating characteristic (ROC) and risk distribution curves suggested this signature was an accurate predictor independent of risk factors. A nomogram incorporating the signature risk score and stage was constructed, and the calibration plot suggested it could accurately predict the survival rate. Compared with normal tissues, tumor tissues had increased mRNA levels of *GNG3* and *PRXL2A* and a reduced mRNA level of *ITIH3*. The knockdown of PRXL2A and GNG3 significantly inhibited the proliferation and colony formation of Ishikawa and AN3CA cells, while the inhibition of PRXL2A expression suppressed xenograft growth. In this study, five estrogen/progesterone-related genes were identified and incorporated into a novel signature, which provided a new classification tool for improved risk assessment and potential molecular targets for EC therapies.

## 1. Introduction

Endometrial Cancer (EC) is one of the three most common cancers of the female reproductive system and there were approximately 417,000 new diagnoses and 97,300 deaths of EC in 2020 worldwide, according to GLOBOCAN statistics [1,2]. Obesity, hypertension and diabetes mellitus resulting from changes in modern lifestyles have gradually increased the incidence of EC over recent decades, seriously endangering women’s health [3,4,5]. EC is predominantly a disease of postmenopausal women, and the previous studies have suggested that the underlying mechanisms of EC occurrence include an imbalance between estrogen and progesterone [6,7,8]. Estrogen mainly regulates EC cells through estrogen receptor α (ERα) or estrogen receptor β (ERβ), and 75–90% of the total incidence of EC is dependent on estrogen [9]. Liu et al. demonstrated that estrogen acts through ERβ to enhance the activation of the NLPR3 inflammasome and promote the progression of EC [10]. TFEB and ERα are highly expressed in tumors and correlated with EC progression; furthermore, ERα is the direct target of TFEB, mediating lipid metabolism in EC [11]. ERα interacts with hnRNP K to regulate endometrial changes during the menstrual cycle and suppresses the malignant behavior of EC [12]. Progesterone and progesterone receptor (PR) can promote the progression of breast cancer, whereas the effect of progesterone is protective against the development of estrogen-driven EC [13,14]. Progesterone can induce EC cell apoptosis via the CACNA2D3/Ca^2+^/p38 MAPK pathway and inhibit Wnt signaling by DKK1 and FOXO1 in Wnt-activated EC cells [15,16]. At present, oral progesterone therapy is by far the most common option for the conservative management of EC. Current research also suggests that ER status is significantly associated with overall survival (OS), and ER and PR expression are independent biomarkers of disease-specific survival [17,18]. The estrogen/progesterone pathway is essential for EC occurrence and development; we aimed to conduct an in-depth exploration of biomarkers or classifiers that can accurately predict the prognosis and therapeutic targets that can be employed for individualized treatment based on this pathway. In addition, the molecular typing of EC based on The Cancer Genome Atlas (TCGA) was reported in 2013 [19], possessing significant value for research and clinical translation. Therefore, it is necessary to conduct research on the classification of the EC based on the estrogen/progesterone pathway.

In this study, we developed a novel signature based on estrogen/progesterone-related genes to assess the prognosis of EC patients in the TCGA database. In addition, we explored the function of abnormal expression of these genes in EC; the genes composing this signature may become potential therapeutic targets in the future.

## 2. Materials and Methods

### 2.1. Data Extraction and Preprocessing

EC patients with complete prognostic data were included in this study. Clinical information and transcriptome data (FPKM values) from 397 EC patients and 23 normal tissues were acquired from the TCGA database. The keyword “estrogen” or “progesterone” was searched in the Molecular Signatures Database [20]. Then, 51 gene sets (Supplementary Materials Table S1) were obtained to construct an estrogen/progesterone-related gene expression matrix.

### 2.2. Heatmap, Gene Set Enrichment Analysis (GSEA), Gene Ontology (GO) Analysis and Kyoto Encyclopedia of Genes and Genomes (KEGG) Pathway Enrichment Analysis

A heatmap was drawn using the “pheatmap” R package. GSEA was performed to explore the differences in the enrichment of the above gene sets between normal and tumor tissues. |Normalized enrichment score (NES)| > 1, nominal (NOM) *p*-value < 0.05 and false discovery rate (FDR) q-value < 0.25 were set as the cutoffs. Estrogen/progesterone-related differentially expressed genes (DEGs) with an FDR < 0.05 were identified via the “limma” R package between normal and tumor tissues, and then these DEGs were analyzed by the “clusterprofiler” R package for GO and KEGG pathway enrichment analyses.

### 2.3. Establishment and Validation of a Novel Estrogen/Progesterone Gene Signature

Univariate and multivariate Cox regression analyses were performed to identify estrogen/progesterone-related DEGs associated with OS (*p* < 0.05), and the filtered mRNAs were classified into risk (hazard ratio (HR) > 1) and protective (0 < HR < 1) types. To predict each individual patient’s survival probability, the risk score was calculated with the following formula:

Risk Score=∑i Coefficient (mRNAi) × mRNAi level (mRNAi)

With the median risk score as the cutoff, the patients were divided into high-risk and low-risk groups. Kaplan–Meier (KM) survival, time-dependent receiver operating characteristic (ROC) and risk distribution analyses were conducted to evaluate the prognostic significance of the signature. The R packages applied were “survival”, “survivalROC” and “pheatmap”.

### 2.4. OS Assessment of the High-Risk and Low-Risk Groups in Different Clinical Subgroups

Based on age, BMI, tumor grade and hypertension status, the patients were divided into age > 50 and age ≤ 50 subgroups, BMI > 30 and BMI ≤ 30 subgroups, Grade 1 + Grade 2 and Grade 3 subgroups, and Hypertension_NO and Hypertension_YES subgroups. Subsequently, KM survival analyses were performed to explore the prognostic differences between high-risk and low-risk patients in the above eight clinical subgroups.

### 2.5. Gene Set Variation Analysis (GSVA), Tumor-Infiltrating Immune Cells Analysis and Nomogram Construction

To determine the correlation between the risk score and biological pathways, we further characterized the corresponding gene expression profiles of EC samples via single-sample gene set enrichment analysis (ssGSEA) using the R package “GSVA”. We obtained an enrichment score corresponding to each function for each sample, and the correlation between these functions and the risk score was further calculated.

With the CIBERSORT algorithm, the RNA-seq data from the TCGA set were applied to calculate the abundance degree of 22 tumor-infiltrating immune cells in each EC sample. The abundance degree difference analysis between the high-risk and low-risk groups was conducted by the “limma” R package.

A nomogram was constructed for predicting survival outcomes (2-year, 5-year and 8-year survival) by the “rms” R package. Calibration plots were drawn to determine the consistency between the actual survival rates and the nomogram-predicted rates.

### 2.6. Clinical Samples

A total of 65 fresh EC samples and 22 normal samples were surgically resected at the Chinese Academy of Medical Sciences and the CAMS & PUMC Medical College. The enrolled cohort contained all 3 grades (grade 1, grade 2 and grade 3) of histopathological differentiation and 4 stages (stage I, stage II, stage III and stage IV) of the EC. None of the patients had received any treatment prior to surgery, and each patient signed informed consent.

### 2.7. Total RNA Extraction

Total RNA was isolated from tissues or cells using the RNApure Tissue & Cell Kit (Cwbiotech, Beijing, China) as described by the manufacturer.

### 2.8. Quantitative Real-Time Polymerase Chain Reaction (qRT–PCR) Assay

Isolated RNA was used as a template for reverse transcription reactions using the HiFiScript cDNA Synthesis Kit (Cwbiotech, Beijing, China). Then qRT–PCR assay was performed using the SYBR^®^ Premix Ex Taq™ (TaKaRa, Shiga, Japan) and the CFX96 Real-Time System (Bio-Rad, Hercules, CA, USA) to detect the relative mRNA levels of *PRXL2A*, *GNG3* and *ITIH3*. The primer sequences are shown in the Supplementary Materials Table S2. The relative mRNA level of the target genes was normalized to an endogenous reference (*GAPDH*). Data were presented as mean ± standard error of mean (SEM).

### 2.9. Western Blotting Assay

SDS-PAGE and western blotting were carried out according to standard protocols. The protein extracts were quantitated using the Pierce™ BCA Protein Assay Kit (Thermo Fisher Scientific, Waltham, MA, USA). Protein samples (20–60 μg) were separated on SDS-PAGE gels and then transferred onto PVDF membranes (Millipore, Bedford, MA, USA). GAPDH was used as an internal control. The blots were blocked and probed with primary antibodies against GAPDH (Proteintech, Wuhan, China), ITIH3 (Proteintech, Wuhan, China) and DYKDDDDK tag (Cell Signaling Technology, Danvers, MA, USA).

### 2.10. Cell Culture, Transfection and Lentiviral Transduction

The human EC cell lines (Ishikawa and AN3CA) were provided by the National Collection of Authenticated Cell Cultures (Shanghai, China). Both cell lines were cultured in Eagle’s minimum essential medium supplemented with 10% fetal bovine serum, 1% nonessential amino acids, 1% sodium pyruvate and 5% CO_2_ at 37 °C. The specific siRNA sequences for the decrease in PRXL2A and GNG3 are provided in Supplementary Materials Table S3. The siRNA was transfected with Lipofectamine 2000 (Invitrogen, San Diego, CA, USA) according to the manufacturer’s instructions, and the final concentration used for gene silencing was 50 nM. Lentiviruses were used to transduce EC cells. The stable cell strains expressing PRXL2A-shRNA (shPRXL2A) or control scrambled shRNA (sh-scramble) were selected using puromycin (1 μg/mL, Gibco) for at least 5 days, and the stable cell strains expressing pLVX-ITIH3-Flag-IRES-Neo or control empty vector were selected using G418 (0.6 mg/mL, Gibco) for at least 1 week. The lentiviral transduction method was described in our previous article [21].

### 2.11. Cell Proliferation, Cell Cycle and Colony Formation Assays

Cells were seeded at a density of 2000 cells per well into 96-well plates. According to the manufacturer’s protocol, cell viability was detected by the Cell Counting Kit-8 (CCK-8) assay (Dojindo, Kumamoto, Japan). An automatic microplate reader (BioTek, Winooski, VT, USA) was used to measure the absorbance at 450 nm. Measurements were taken every 24 h for 6 consecutive days. Cells were seeded in a 6-well plate at a density of 4 × 10^5^ cells per well and incubated at 37 °C for 24 h. Cells were treated with siRNA for 24 h, collected by trypsinization and fixed in ice-cold 70% ethanol overnight at 4 °C. The cell cycle detection kit (KeyGen Biotech, Nanjing, China) and the BDTM LSRIII flow cytometer (BD Biosciences, San Jose, CA, USA) were used to analyze the cell cycle distribution. To evaluate the clonality of cells in vitro, siRNA-treated cells were seeded in 6-well culture plates (500 cells/plate). The cells were allowed to grow for 10–14 days to form colonies, then fixed with methanol and stained with crystal violet. Data of the cell proliferation, cell cycle and colony formation assays were presented as mean ± SEM. 

### 2.12. Xenograft Model Assay

Four-week-old female BALB/c nude mice (HFK Bioscience, Beijing, China) were equally divided into two groups (5 mice/group) and given a subcutaneous injection of 2 × 10^6^ AN3CA cells in which PRXL2A was either stably knocked out or left intact. The tumor size and body weight were measured every 4 days, and tumor volume was calculated by the following formula: volume = length × width^2^ × π/6. After 13 days from the first measurement, the mice were sacrificed and weight of the xenografts was measured.

### 2.13. Statistical Analysis

All statistical analyses were performed using SPSS 22.0 software (SPSS Inc. Chicago, IL, USA) and R software. A probability value of *p* < 0.05 was considered significant. Student’s *t* test was used to determine the significance of differences between two groups, and ANOVA was used for comparisons among more than two groups.

## 3. Results

### 3.1. Identification and Enrichment Analysis of Estrogen/Progesterone-Related DEGs

**Figure 1:**
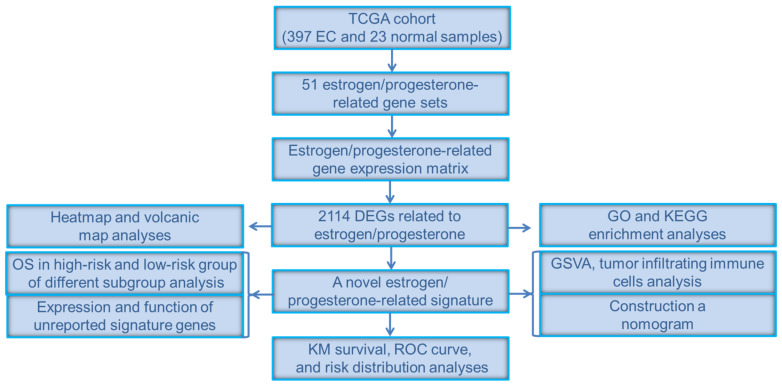
Flow chart of the study.

**Figure 2:**
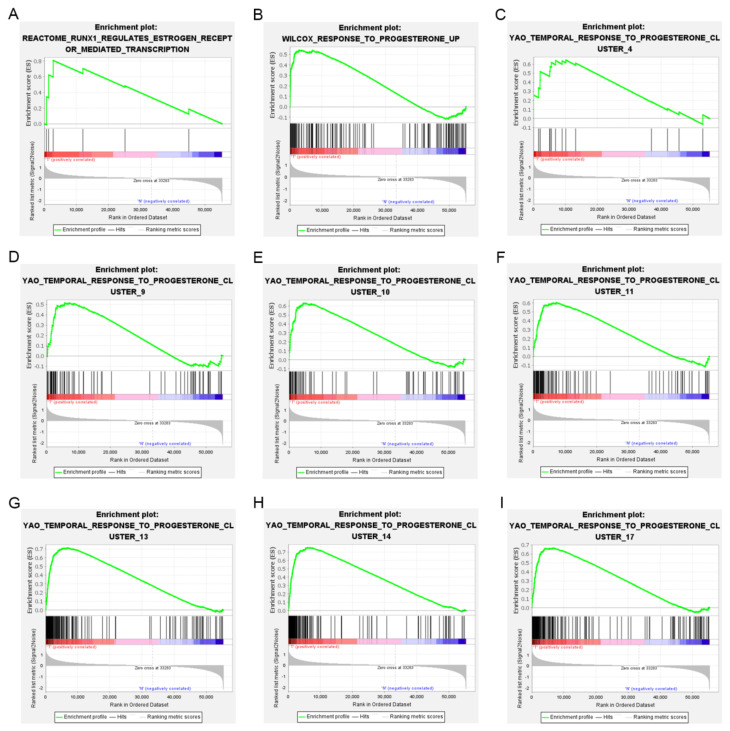
Nine estrogen/progesterone-related gene sets were highly enriched in EC. (**A**) REACTOME_RUNX1_REGULATES_ESTROGEN_RECEPTOR_MEDIATED_TRANSCRIPTION. (**B**) WILCOX_RESPONSE_TO_PROGESTERONE_UP. (**C**) YAO_TEMPORAL_ RESPONSE_TO_PROGESTERONE_CLUSTER_4. (**D**) YAO_TEMPORAL_RESPONSE_ TO_PROGESTERONE_CLUSTER_9. (**E**) YAO_TEMPORAL_RESPONSE_TO_ PROGES TERONE_CLUSTER_10. (**F**) YAO_TEMPORAL_RESPONSE_TO_ PROGESTERONE_ CLUSTER_11. (**G**) YAO_TEMPORAL_RESPONSE_TO_PROGESTERONE_CLUSTER_13. (**H**) YAO_TEMPORAL_RESPONSE_TO_PROGESTERONE_CLUSTER_14. (**I**) YAO_TEMPORAL_ RESPONSE_TO_PROGESTERONE_CLUSTER_17.

In order to show the idea of the research more intuitively, we generated a flow chart in this study (Figure 1). We obtained clinical and transcriptome data of 397 tumor and 23 normal samples from the TCGA database. Combining TCGA transcriptome data and 51 estrogen/progesterone-related gene sets, we constructed an estrogen/progesterone-related gene expression matrix. GSEA was performed to identify estrogen/progesterone-related biological processes between normal and EC tissues. It confirmed that approximately 18% (9/51) of the gene sets were highly enriched in EC (Figure 2), suggesting that estrogen/progesterone-related genes played an essential role in EC.

**Figure 3:**
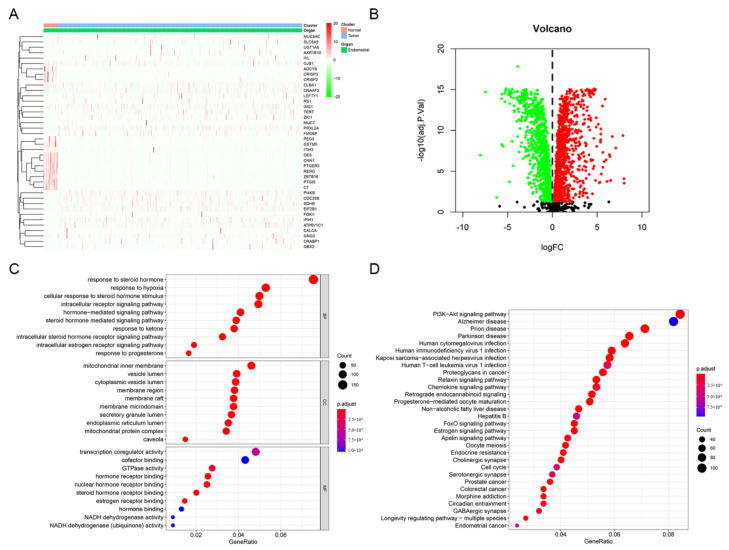
Identification and enrichment analysis of estrogen/progesterone-related DEGs. (**A**) Heatmap of estrogen/progesterone-related representative DEGs between tumor and normal tissues. (**B**) Volcano plot of estrogen/progesterone-related DEGs. (**C**) The results of GO enrichment analysis of estrogen/progesterone-related DEGs are shown by a bubble chart: molecular function (MF), biological process (BP), and cellular component (CC). (**D**) KEGG enrichment analysis of estrogen/progesterone-related DEGs.

A total of 2114 DEGs related to estrogen/progesterone were identified between tumor and normal tissues, including 1139 upregulated genes and 975 downregulated genes. The heatmap and volcanic map of DEGs are shown in Figure 3A,B. Furthermore, GO and KEGG pathway enrichment analyses were carried out to explore the signaling pathways and biological processes in which these DEGs might be involved. The GO enrichment results indicated that the enriched biological process (BP) terms included the intracellular estrogen receptor signaling pathway and response to progesterone, while the molecular function (MF) terms involved estrogen receptor binding (Figure 3C). Interestingly, KEGG enrichment results indicated these DEGs were enriched not only in the estrogen signaling pathway and progesterone-mediated oocyte maturation but also in the PI3K-Akt signaling pathway, chemokine signaling, the cell cycle and EC (Figure 3D). It suggested that estrogen/progesterone-related DEGs might regulate the occurrence and development of EC by participating in biological pathways such as immunity, cell cycle regulation and tumor signaling activation.

### 3.2. Construction of a Signature Incorporating Five Estrogen/Progesterone-Related Genes

**Figure 4:**
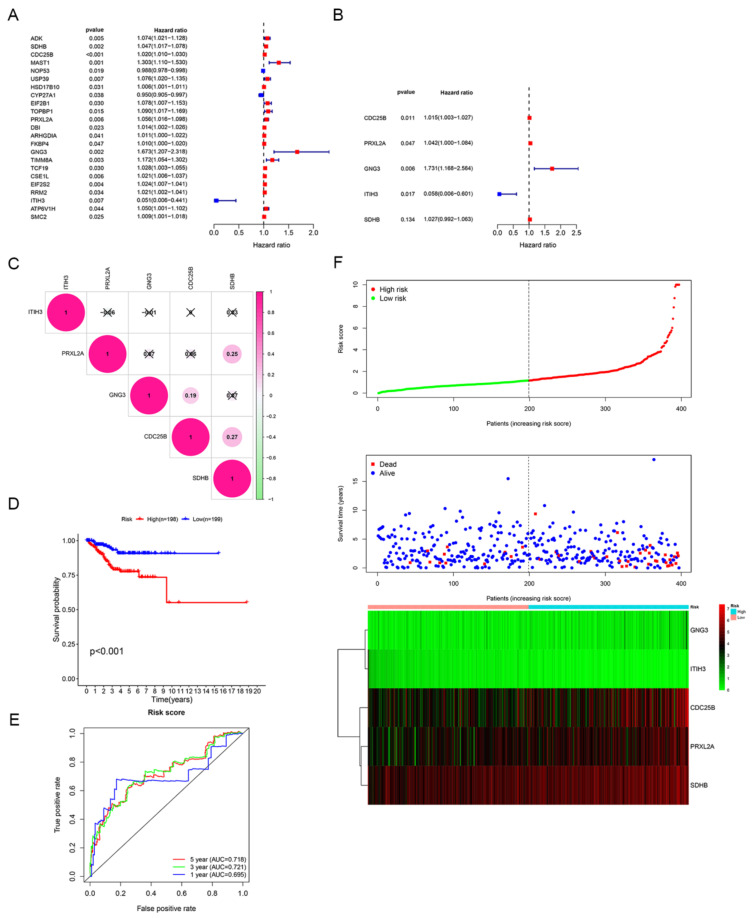
Identification of the estrogen/progesterone-related signature. (**A**,**B**) Five estrogen/progesterone-related genes were screened to identify a signature according to univariate (**A**) and multivariate Cox regression analyses (**B**). (**C**) Correlation analysis of the mRNA levels of the signature genes. (**D**) KM survival curves of the high-risk and low-risk groups. (**E**) ROC curve analysis was performed to assess the accuracy of the signature for predicting 1-, 3- and 5-year survival. (**F**) Risk score distribution, patient survival status and signature gene expression profiles.

Based on 2114 candidate DEGs, univariate and multivariate Cox regression analyses were performed to construct a risk signature. A forest plot of HRs showed that *CDC25B*, *PRXL2A*, *GNG3* and *SDHB* were risk factors related to OS (HR > 1) and *ITIH3* was a protective factor for OS (HR < 1) (Figure 4A,B). Finally, a five−gene signature related to estrogen/progesterone was established. The formula for the risk score was as follows: risk score = (0.0150 × *CDC25B* mRNA level) + (0.0407 × *PRXL2A* mRNA level) + (0.5485 × *GNG3* mRNA level) + (−2.8448 × *ITIH3* mRNA level) + (0.0267 × *SDHB* mRNA level).

Furthermore, we performed an in-depth analysis of gene alterations, mRNA levels and prognostic correlations of five model genes. Based on 383 patients on the cBioPortal website (patients from TCGA), the genetic alterations of *ITIH3*, *PRXL2A*, *CDC25B*, *GNG3* and *SDHB* were 8.36%, 4.70%, 4.44%, 2.35% and 2.35%, respectively (Supplementary Figure S1A). By conducting between-groups and paired analyses of normal and tumor tissues, the results showed that the *ITIH3* mRNA level was downregulated while the other four signature genes were all upregulated in tumors (Supplementary Figure S1B–G). Correspondingly, a high mRNA level of *ITIH3* (*p* < 0.01) was positively correlated with longer OS, but high mRNA levels of *CDC25B* (*p* < 0.05), *GNG3* (*p* < 0.001), *PRXL2A* (*p* < 0.05) and *SDHB* (*p* < 0.05) were negatively correlated with longer OS (Supplementary Figure S1H–L). The correlation analysis showed that the absolute values of R were consistently less than 0.3, indicating that the five model genes were independent of each other (Figure 4C). Based on the median risk score, patients were divided into high-risk and low-risk groups. Patients with high risk scores had dramatically worse OS than those with low risk scores (*p* < 0.001; Figure 4D). To investigate the diagnostic accuracy of the signature, we calculated the time-dependent area under the ROC curve (AUC). The AUCs for predicting 1-year, 3-year, and 5-year survival were 0.695, 0.721, and 0.718, respectively (Figure 4E). The risk score increased when the mRNA levels of *CDC25B*, *PRXL2A*, *GNG3* and *SDHB* were upregulated and when the mRNA level of *ITIH3* was gradually downregulated; additionally, the number of deaths increased as the risk score increased (Figure 4F). These results suggested that this signature could effectively predict patient survival.

### 3.3. OS Assessment of the High-Risk and Low-Risk Groups in Different Clinical Subgroups

**Figure 5:**
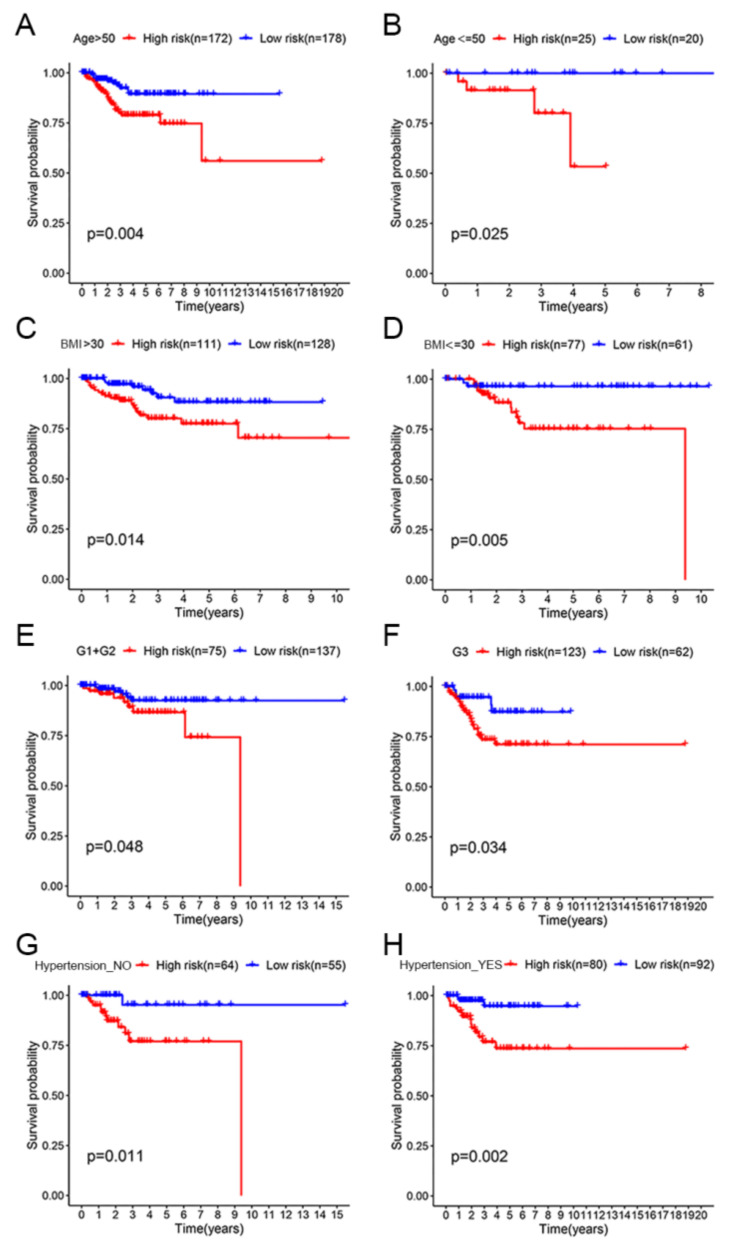
OS assessment of the high-risk and low-risk groups in different clinical subgroups. (**A**) Age > 50 subgroup. (**B**) Age ≤ 50 subgroup. (**C**) BMI > 30 subgroup. (**D**) BMI ≤ 30 subgroup. (**E**) Grade 1 + Grade 2 subgroup. (**F**) Grade 3 subgroup. (**G**) Hypertension_NO subgroup. (**H**) Hypertension_YES subgroup.

To clarify the applicability of the prognostic signature in EC patients, KM survival analyses were performed to explore the prognostic differences between high-risk and low-risk patients in the eight clinical subgroups. The OS of low-risk patients was significantly longer than that of high-risk patients in the age >50 subgroup (*p* < 0.01; Figure 5A). We obtained consistent conclusions in the age ≤ 50 subgroup (*p* < 0.05; Figure 5B), BMI > 30 subgroup (*p* < 0.05; Figure 5C), BMI ≤ 30 subgroup (*p* < 0.01; Figure 5D), Grade 1 + Grade 2 subgroup (*p* < 0.05; Figure 5E), Grade 3 subgroup (*p* < 0.05; Figure 5F), Hypertension_NO subgroup (*p* < 0.05; Figure 5G) and Hypertension_YES group (*p* < 0.01; Figure 5H). In all eight clinical subgroups, a better prognosis was demonstrated in the low-risk group than in the high-risk group, which indicated that our prognostic signature can be applied to predict the prognosis of different clinical types of EC patients.

### 3.4. GSVA and Tumor-Infiltrating Immune Cells Analysis of the Signature between the High-Risk and Low-Risk Groups

**Figure 6:**
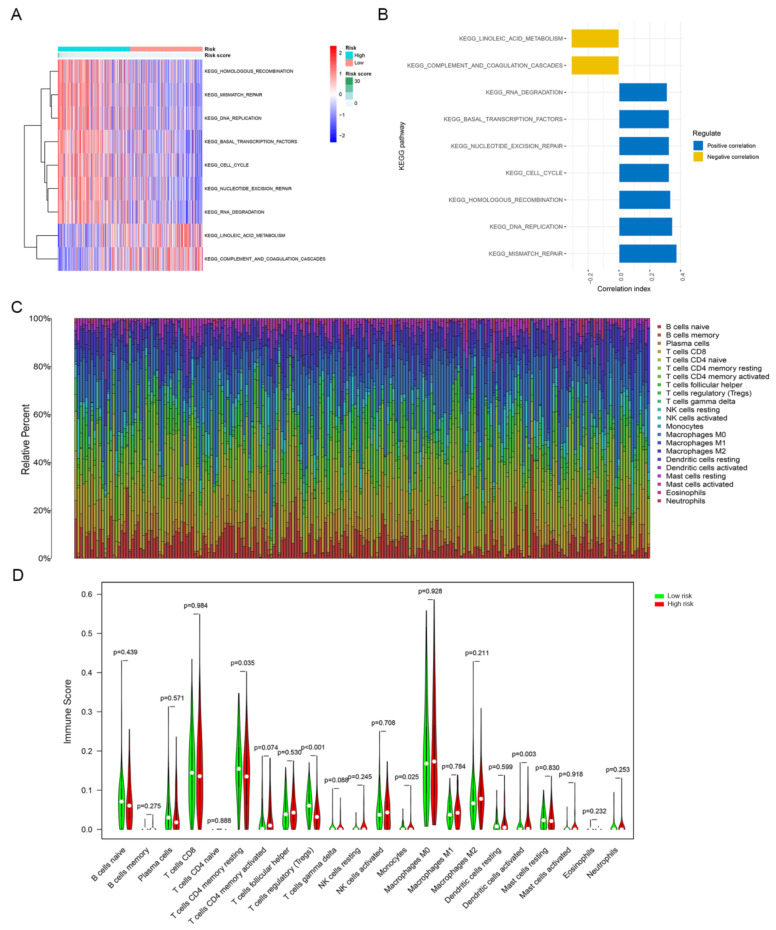
GSVA and tumor-infiltrating immune cells analysis of the signature between the high-risk and low-risk groups. (**A**) GSVA revealed KEGG pathways associated with the risk score, and the risk score decreases from left to right. (**B**) The top 9 signature-related KEGG pathways. (**C**) Tumor-infiltrating immune cells proportions in each sample. (**D**) Different infiltration levels of 22 tumor-infiltrating immune cells between the high-risk and low-risk groups.

In order to explore the relationship between the risk score and biological function in different samples, ssGSEA was performed using the “GSVA” R package. Enrichment analysis revealed the top 9 KEGG pathways closely associated with the risk score (Figure 6A). KEGG_RNA_DEGRADATION, KEGG_BASAL_TRANSCRIPTION_FACTORS, KEGG _NUCLEOTIDE_EXCISION_REPAIR, KEGG_CELL_CYCLE, KEGG_HOMO LOGOUS _RECOMBINATION, KEGG_DNA_REPLICATION and KEGG_MISMATCH _REPAIR were positively related to the risk score. KEGG_LINOLEIC_ACID_ METABOLISM and KEGG_COMPLEMENT_AND_COAGULATION_CASCADES were negatively correlated with the risk score (Figure 6B). Interestingly, we found that the KEGG pathway enriched for estrogen/progestin-related DEGs included cell cycle in Figure 3D, and here the KEGG pathway significantly associated with the risk score also included the cell cycle.

We determined the infiltration levels of 22 major tumor-infiltrating immune cells in each sample via the CIBERSORT algorithm (Figure 6C). The infiltration levels of four types of tumor-infiltrating immune cells were different between the high-risk and low-risk groups. The abundance levels of resting memory CD4^+^ T cells (*p* < 0.05), regulatory T cells (*p* < 0.001) and monocytes (*p* < 0.05) were higher in the low-risk group than in the high-risk group. In contrast, the abundance level of activated dendritic cells was remarkably higher in the high-risk group than in the low-risk group (*p* < 0.01; Figure 6D).

### 3.5. Construction of a Nomogram Incorporating the Signature for Predicting OS

**Figure 7:**
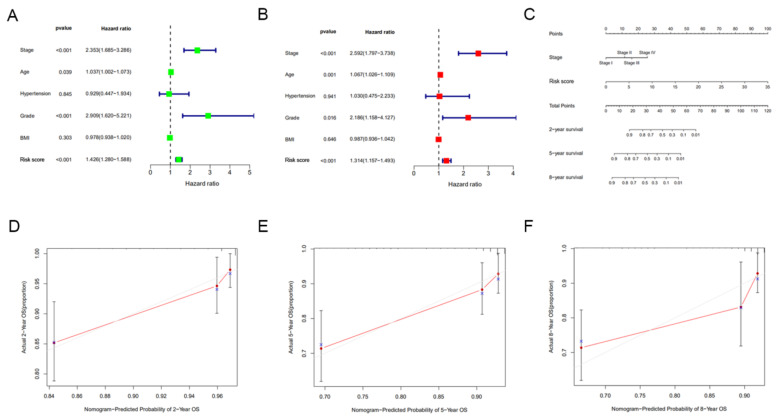
Construction and evaluation of a nomogram incorporating the signature. (**A**) Univariate Cox regression analysis of clinical characteristics and risk score. (**B**) Multivariate Cox regression analysis of clinical characteristics and risk score. (**C**) A nomogram for predicting the 2-, 5- and 8-year survival rates of EC patients was established. (**D**–**F**) The calibration curves showed the actual rate versus predicted probability of 2- (**D**), 5- (**E**) and 8-year (**F**) survival.

Univariate and multivariate Cox regression analyses were performed to assess the independent prognostic value of clinicopathological parameters (stage, age, hypertension, grade and BMI) and risk score derived from the TCGA set. The results showed that the risk score was an independent prognostic factor for OS in EC. Consistent with clinical reports, the stage was also an independent prognostic factor (Figure 7A,B). The stage and risk score were incorporated into the nomogram to make it more intuitive and effective (Figure 7C). The calibration curves for 2-year, 5-year and 8-year survival rates were highly similar to the trend of the standard curve (Figure 7D–F). These results demonstrated this nomogram built based on the risk score can accurately predict the short-, medium-, and long-term survival of patients.

### 3.6. Expression and Functional Analysis of GNG3, ITIH3 and PRXL2A in EC

**Figure 8:**
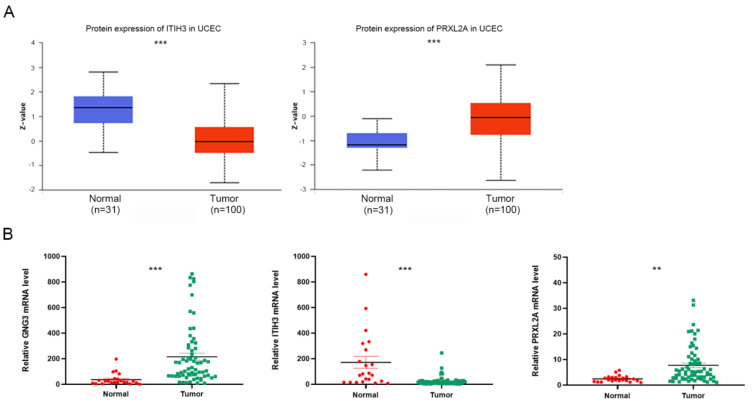
Expression of GNG3, ITIH3 and PRXL2A in tumor and normal tissues. (**A**) Differential protein expression of ITIH3 and PRXL2A between tumor and normal tissues in the UALCAN database. (**B**) qRT–PCR analyses of *GNG3*, *ITIH3* and *PRXL2A* mRNA levels between tumor and normal tissues. **, *p* < 0.01; ***, *p* < 0.001.

The expression and roles of CDC25B and SDHB in EC have been reported in the literature. CDC25B expression is elevated in EC cells compared to normal cells, while knockdown of CDC25B expression can induce G2/M cell-cycle arrest and inhibit cell proliferation in KLE and HEC1B cells [22,23]. An estrogen-mediated decrease in SDHB can lead to the accumulation of succinic acid, which regulates the proliferation of EC cells and the growth of xenograft tumors [24]. In our study, we investigated the expression and function of GNG3, ITIH3 and PRXL2A, which have not yet been reported in EC. According to the UALCAN website EC study cohort, tumor tissues have lower expression of ITIH3 protein (*p* < 0.001) and higher expression of PRXL2A protein (*p* < 0.001) than normal tissues (Figure 8A). We could not retrieve the protein expression of GNG3 from the UALCAN website; perhaps the coverage of the proteome was incomplete [25]. Then, we performed qRT–PCR to compare the mRNA levels of *GNG3*, *ITIH3* and *PRXL2A* in 65 EC and 22 normal tissue samples. The mRNA levels of *GNG3* (*p* < 0.001) and *PRXL2A* (*p* < 0.01) were upregulated in EC, while the mRNA level of ITIH3 (*p* < 0.001) was downregulated, consistent with the mRNA changes of model genes in the TCGA cohort (Figure 8B). These UALCAN analysis results and confirmatory experiments revealed consistent changes in model genes, which justified our TCGA bioinformatics analysis.

**Figure 9:**
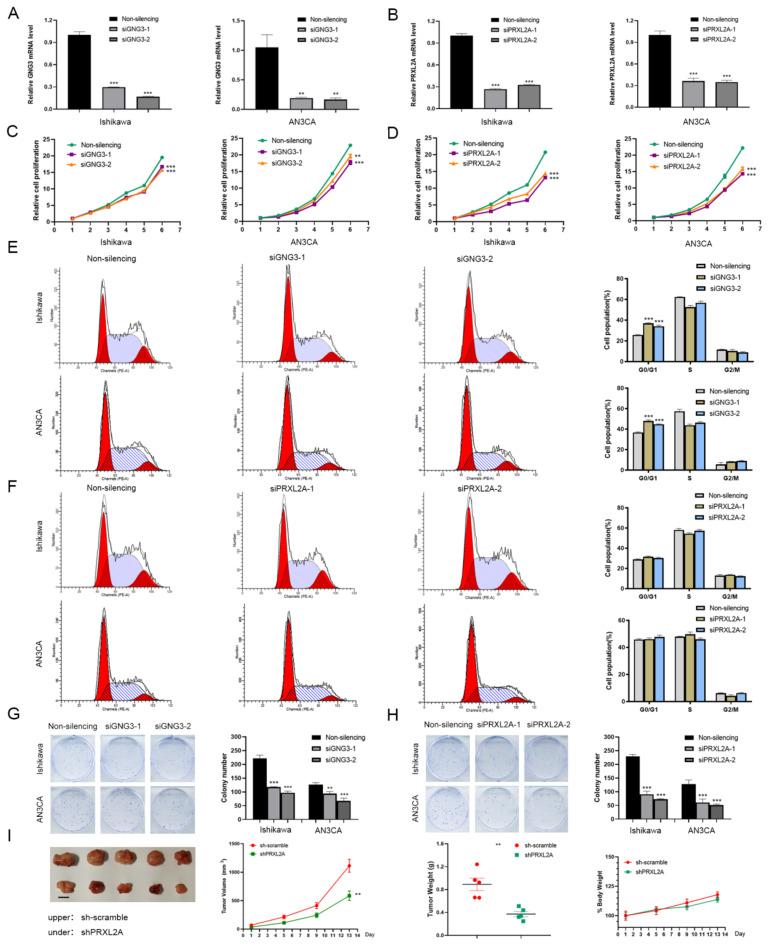
Functional analysis of GNG3 and PRXL2A in EC. (**A**,**B**) The specific siRNAs targeting GNG3 (**A**) and PRXL2A (**B**) successfully silenced their expression in EC cells. (**C**,**E**,**G**) Cell proliferation (**C**), cell cycle distribution (**E**) and colony-forming ability (**G**) were detected when GNG3 was knocked down. (**D**,**F**,**H**) Cell proliferation (**D**), cell-cycle distribution (**F**) and colony-forming ability (**H**) were detected when PRXL2A was knocked down. (**I**) Xenograft tumors, volume change curve of xenograft tumors, weights of xenograft tumors and weights of nude mice. Data are presented as mean ± SEM (*n* = 5). Body weight is expressed as a percentage relative to the initial weight. **, *p* < 0.01; ***, *p* < 0.001.

We knocked down protein expression in Ishikawa and AN3CA cells using specific siRNAs to clarify the functional roles of GNG3 and PRXL2A (Figure 9A,B). Through CCK-8 detection, we found that downregulation of GNG3 or PRXL2A can inhibit the proliferation of EC cells (Figure 9C,D). Furthermore, suppression of GNG3 dramatically induced cell cycle arrest in the G0/G1 phase, whereas PRXL2A did not affect the cell cycle (Figure 9E,F). Knockdown of GNG3 or PRXL2A expression significantly inhibited the colony-forming capacity of EC cells (Figure 9G,H). Since ITIH3 is expressed at low levels in EC samples, overexpression of ITIH3 did not affect the proliferative activity of cells (Supplementary Figure S2A–D). In the subsequent experiment, we constructed a cell line in which PRXL2A was stably knocked out by a lentiviral vector, and then we performed xenograft experiments. The nude mice bearing xenograft tumors were divided into control sh-scramble and shPRXL2A groups. The changes in tumor volume and weight are shown in Figure 9I. This result indicated that the stable knockout of PRXL2A significantly inhibited tumor growth.

## 4. Discussion

With the rapid development of high-throughput sequencing technology, TCGA has integrated genomics, transcriptomics, gene copy number and clinical information to study and classify four different molecular subtypes: (I) POLE (ultramutated), (II) MSI (hypermutated), (III) copy-number low and (IV) copy-number high [19]. Approximately 10% of EC patients carry one of these pathogenic POLE mutations, the 5-year progression-free survival of patients with POLE pathogenic mutations was 97.7% and patients with POLE mutations showed significantly better prognoses than the patients without POLE mutations in progression-free survival [26]. Microsatellite instability-high (MSI-H) is a biomarker for response to immunity therapy, and approximately one-third of EC patients exhibit an MSI-H phenotype [27]. Dostarlimab demonstrates durable antitumor activity (ORR: 43.5%) in dMMR/MSI-H EC with a manageable safety profile, which is a humanized monoclonal antibody that binds with high affinity to PD-1 [28]. The phase II KEYNOTE-158 study evaluated the anti-tumor activity and safety of Pembrolizumab. Among 49 EC patients with dMMR/MSI-H who were enrolled in the study, the ORR was 57.1% (95% CI, 42.2–71.2%), and 8 patients (16.3%) had a complete response [29]. Molecular typing based on TCGA cohort can not only predict prognosis but also guide precise treatment. In 2020, the ESGO-ESTRO-ESP guidelines were updated, sharing the conclusions of TCGA molecular classification; the new guidelines encouraged molecular typing of all EC patients [30]. This development heralded the translational application of molecular typing to the clinic, which greatly encouraged us to use the TCGA database for tumor molecular typing. In this case, classification of EC based on characteristic gene and patient prognostic information might provide a reasonable prediction of patient disease risk.

Estrogen mainly promotes the proliferation of EC cells, and progesterone inhibits their proliferation. The dynamic imbalance between estrogen and progesterone is an important factor in accelerating tumor formation [31]. For patients who wish to preserve their fertility or who do not meet surgical guidelines, progestins (synthetic progestogens) are given as the main course of treatment. Considering that estrogen, progestin and molecules related to their pathways can play important regulatory roles in EC, we took this as an entry point and incorporated data from the TCGA database to develop a classifier that could accurately predict prognostic risk in this study. We downloaded transcriptomic and clinical information from TCGA to screen 2114 estrogen/progesterone-related DEGs between EC and normal tissues, and five genes (*CDC25B*, *GNG3*, *ITIH3*, *PRXL2A* and *SDHB*) with prognostic value for OS were identified through univariate and multivariate Cox proportional-hazards regression models. Based on these five genes, we constructed a gene signature for predicting the prognosis of patients. Patients in the low-risk group had a significantly better prognosis than those in the high-risk group. The ROC curve and risk distribution analyses showed that this risk signature was an accurate predictor of OS. Survival analysis in eight clinical subgroups showed that patients in the high-risk group based on the signature all had better prognosis than those in the low-risk group. Finally, we confirmed that the risk score was an independent risk factor and constructed a novel nomogram to predict the 2-year, 5-year and 8-year survival rates of EC patients.

The signature identified in this study can not only serve as a classifier to accurately predict patient prognosis but also identify potential candidate targets for targeted therapy. In this study, we found that ITIH3 did not affect EC cell proliferation, but knockdown of GNG3 and PRXL2A both inhibited the proliferation activity and colony formation of EC cells, and further stable knockdown of PRXL2A significantly suppressed the growth of xenografts. High PRXL2A expression is positively correlated with poor prognosis of oral squamous cell carcinoma, and a decrease in PRXL2A expression can significantly inhibit cell proliferation, migration and invasion [32]. GNG3 was found to be a hub gene via CytoHubba in Cytoscape, and its main functions were to participate in the cell cycle and p53 signaling pathway in glioblastoma [33]. The other two model genes, *SDHB* and *CDC25B,* have been studied in depth; *SDHB* participates in the tricarboxylic acid cycle, and mutations in this gene are closely related to the pathogenesis of a variety of tumors [34]. *SDHB*-deficient tumor cells undergo a switch in cell metabolism, gaining oxidative phosphorylation and glycolysis capacity and then adapting to changes in the tumor microenvironment [35]. *CDC25B* is currently considered a promising therapeutic target. *CDC25B* mRNA levels are significantly elevated in esophageal squamous cell carcinoma tissues, and higher levels of CDC25B are present in sera from tumors than in sera from healthy control subjects [36,37]. The mRNA and protein levels of *CDC25B* in liver tumors are higher than those in normal tissues, and specific siRNA-targeting CDC25B inhibited liver cancer cell growth in vitro [38]. CDC25B is overexpressed in ovarian cancer and is associated with poor patient prognosis; the CDC25B inhibitor WG-391D can significantly inhibit the malignant proliferation of ovarian cancer cells and the growth of transplanted tumors [39]. Naphthylphenylketone and naphthylphenylamine derivatives that function as CDC25B phosphatase inhibitors potently inhibited cell proliferation and colony formation, inducing G2/M-phase cell-cycle arrest in melanoma cell lines [40]. CDC25B has been widely confirmed to promote cancer by activating Cyclin/CDK [41,42]. It is a member of the CDC25 family, which is a highly conserved, dual-specificity tyrosine phosphatase responsible for regulating cell cycle transition, DNA replication and mitosis. In addition to its cell cycle role, CDC25B also acts as a steroid receptor coactivator [43]. When coexpressed with ER, CDC25B can coactivate ER-dependent reporter genes in the presence of estradiol. Coactivation by CDC25B regulates glucocorticoid receptors, progesterone receptors and androgen receptors [44]. Several CDC25B inhibitors have been developed, and we look forward to their early clinical application.

This study identified and validated a novel estrogen/progesterone-related signature based on bioinformatics analysis and experiments. On the one hand, the KEGG enrichment results of estrogen/progesterone-related DEGs, the risk score GSVA results and the signature gene cell experiment results were all closely related to cell cycle regulation. On the other hand, the TCGA database, UALCAN database and qRT-PCR experiments all showed that ITIH3 was downregulated and PRXL2A was upregulated in tumors compared with normal tissues. This not only demonstrates that our bioinformatics analysis is reasonable, but also that the experiments successfully validate the database analysis results. In addition, we not only identified a five−gene signature that could predict patient survival, but also constructed a nomogram based on the signature that could accurately predict the short-, medium-, and long-term survival of patients. Therefore, this study can provide a good strategy for patient survival prediction, which has important clinical application value. Furthermore, we also explored the function of signature genes in EC, which provides candidate molecular targets for the development of EC-targeted therapeutic drugs. However, this study also has some limitations. First, the risk signature needs to be further validated in a multicenter clinical cohort. Second, the specific mechanism of signature genes in EC still needs to be further explored.

## 5. Conclusions

Our research identified a novel estrogen/progesterone-related gene signature and constructed an effective nomogram for the OS prediction of EC patients to guide individualized prognostic prediction. It suggested that the genes composing the signature (such as *PRXL2A* and *GNG3*) are promising therapeutic targets, which could serve as a foundation for the development of targeted drugs for EC.

## Data Availability

The data and materials can be obtained from the first author and corresponding author.

## Supplementary Materials

The following are available online at https://www.mdpi.com/article/10.3390/jpm12060914/s1, Figure S1: The expression and survival analyses of five signature genes in EC and normal tissues, Figure S2: The cell growth curve was measured after overexpression of ITIH3, Table S1: Names of the estrogen/progesterone-related genes sets, Table S2: Primers for qRT-PCR analysis, Table S3: Human specific siRNA.

## Abbreviations

EC: Endometrial cancer; ERα: estrogen receptor α; ERβ: estrogen receptor β; PR: progesterone receptor; OS: overall survival; TCGA: The Cancer Genome Atlas; GSEA: gene set enrichment analysis; GO: Gene Ontology; KEGG: Kyoto Encyclopedia of Genes and Genomes; FDR: false discovery rate; DEGs: differentially expressed genes; HR: hazard ratio; KM: Kaplan-Meier; ROC: receiver operating characteristic; GSVA: gene set variation analysis; ssGSEA: single-sample gene set enrichment analysis; qRT-PCR: quantitative real-time polymerase chain reaction; SEM: standard error of mean; CCK-8: Cell Counting Kit-8; MF: molecular function; BP: biological process; CC: cellular component; AUC: area under the ROC curve; MSI-H: microsatellite instability-high.
