# Supplementary material for: Comprehensive Analysis and Experimental Validation of a Novel Estrogen/Progesterone-Related Prognostic Signature for Endometrial Cancer
Source: J Pers Med. 2022 May 31;12(6):914. doi: 10.3390/jpm12060914 (PMC9225250; doi:10.3390/jpm12060914)
Supplement: Supplementary file 1 [file jpm-12-00914-s001.zip › jpm-1652711-supplementary.pdf]

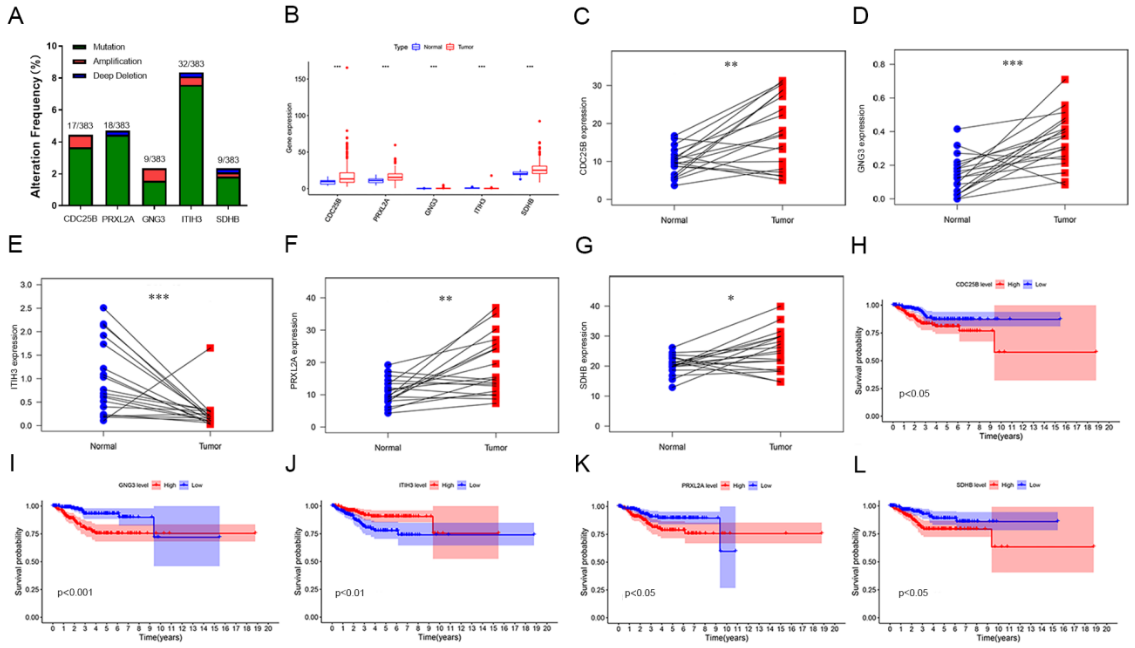

Supplementary Figure S1. The expression and survival analyses of five signature genes in EC and normal tissues. A. Genetic alterations of *CDC25B*, *PRXL2A*, *GNG3*, *ITIH3* and *SDHB*. B. Differential mRNA levels between EC and normal tissues. C-G. The mRNA levels of *CDC25B* (C), *GNG3* (D), *ITIH3* (E), *PRXL2A* (F) and *SDHB* (G) in paired EC and normal tissues. H-L. KM survival curves of patients stratified by *CDC25B* (H), *GNG3* (I), *ITIH3* (J), *PRXL2A* (K) and *SDHB* (L) mRNA levels. *, $p < 0.05$; **, $p < 0.01$; ***, $p < 0.001$.

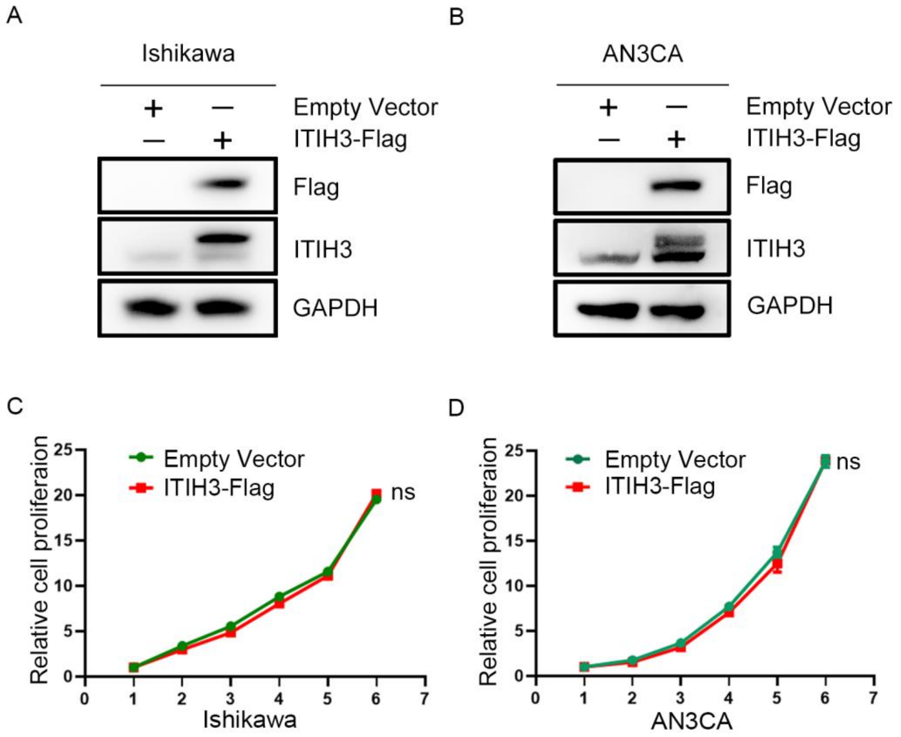

Supplementary Figure S2. The cell growth curve was measured after overexpression of ITIH3. A-B. Western blot analysis of ITIH3 expression in Ishikawa (A) and AN3CA (B) cells transfected with pLVX-ITIH3-Flag-IRES-Neo or an empty vector. C-D. Cell proliferation was detected by the CCK-8 assay in Ishikawa (C) and AN3CA (D) cells. ns, no significance.

Table S1: Names of the estrogen/progesterone-related genes sets

GO_ESTROGEN_2_HYDROXYLASE_ACTIVITY
GO_ESTROGEN_16_ALPHA_HYDROXYLASE_ACTIVITY
GO_ESTROGEN_RECEPTOR_ACTIVITY
GO_ESTROGEN_RECEPTOR_BINDING
GO_INTRACELLULAR_ESTROGEN_RECEPTOR_SIGNALING_PATHWAY
GO_NEGATIVE_REGULATION_OF_INTRACELLULAR_ESTROGEN_RECEPTOR_SIGNALING_PATHWAY
GO_POSITIVE_REGULATION_OF_INTRACELLULAR_ESTROGEN_RECEPTOR_SIGNALING_PATHWAY
GO_PROGESTERONE_RECEPTOR_SIGNALING_PATHWAY
GO_REGULATION_OF_INTRACELLULAR_ESTROGEN_RECEPTOR_SIGNALING_PATHWAY
GO_RESPONSE_TO_PROGESTERONE
GSE22025_PROGESTERONE_VS_TGFB1_AND_PROGESTERONE_TREATED_CD4_TCELL_DN
GSE22025_PROGESTERONE_VS_TGFB1_AND_PROGESTERONE_TREATED_CD4_TCELL_UP
GSE22025_TGFB1_VS_TGFB1_AND_PROGESTERONE_TREATED_CD4_TCELL_DN
GSE22025_TGFB1_VS_TGFB1_AND_PROGESTERONE_TREATED_CD4_TCELL_UP
GSE22025_UNTREATED_VS_PROGESTERONE_TREATED_CD4_TCELL_DN
GSE22025_UNTREATED_VS_PROGESTERONE_TREATED_CD4_TCELL_UP
GSE22025_UNTREATED_VS_TGFB1_AND_PROGESTERONE_TREATED_CD4_TCELL_DN
GSE22025_UNTREATED_VS_TGFB1_AND_PROGESTERONE_TREATED_CD4_TCELL_UP
HP_ABNORMAL_CIRCULATING_ESTROGEN_LEVEL
HP_DECREASED_CIRCULATING_PROGESTERONE
KEGG_PROGESTERONE_MEDIATED_OOCYTE_MATURATION
MALIK_REPRESSED_BY_ESTROGEN
REACTOME_ESTROGEN_BIOSYNTHESIS
REACTOME_EXTRA_NUCLEAR_ESTROGEN_SIGNALING
REACTOME_RUNX1_REGULATES_ESTROGEN_RECEPTOR_MEDIATED_TRANSCRIPTION
STEIN_ESTROGEN_RESPONSE_NOT_VIA_ESRRA
WILCOX_RESPONSE_TO_PROGESTERONE_DN
WILCOX_RESPONSE_TO_PROGESTERONE_UP
WP_ESTROGEN_METABOLISM
WP_ESTROGEN_RECEPTOR_PATHWAY
WP_ESTROGEN_SIGNALING_PATHWAY
YAO_HOXA10_TARGETS_VIA_PROGESTERONE_DN
YAO_HOXA10_TARGETS_VIA_PROGESTERONE_UP
YAO_TEMPORAL_RESPONSE_TO_PROGESTERONE_CLUSTER_0
YAO_TEMPORAL_RESPONSE_TO_PROGESTERONE_CLUSTER_1
YAO_TEMPORAL_RESPONSE_TO_PROGESTERONE_CLUSTER_2
YAO_TEMPORAL_RESPONSE_TO_PROGESTERONE_CLUSTER_3
YAO_TEMPORAL_RESPONSE_TO_PROGESTERONE_CLUSTER_4
YAO_TEMPORAL_RESPONSE_TO_PROGESTERONE_CLUSTER_5
YAO_TEMPORAL_RESPONSE_TO_PROGESTERONE_CLUSTER_6
YAO_TEMPORAL_RESPONSE_TO_PROGESTERONE_CLUSTER_7

YAO_TEMPORAL_RESPONSE_TO_PROGESTERONE_CLUSTER_8
YAO_TEMPORAL_RESPONSE_TO_PROGESTERONE_CLUSTER_9
YAO_TEMPORAL_RESPONSE_TO_PROGESTERONE_CLUSTER_10
YAO_TEMPORAL_RESPONSE_TO_PROGESTERONE_CLUSTER_11
YAO_TEMPORAL_RESPONSE_TO_PROGESTERONE_CLUSTER_12
YAO_TEMPORAL_RESPONSE_TO_PROGESTERONE_CLUSTER_13
YAO_TEMPORAL_RESPONSE_TO_PROGESTERONE_CLUSTER_14
YAO_TEMPORAL_RESPONSE_TO_PROGESTERONE_CLUSTER_15
YAO_TEMPORAL_RESPONSE_TO_PROGESTERONE_CLUSTER_16
YAO_TEMPORAL_RESPONSE_TO_PROGESTERONE_CLUSTER_17

Table S2: Primers for qRT-PCR analysis.

Gene	Primer	Sequence (5’ to 3’)
GAPDH	Forward	CAGCCTCAAGATCATCAGCA
	Reverse	TGTGGTCATGAGTCCTTCCA
PRXL2A	Forward	GGAAACCTGGAAGGAGAAGG
	Reverse	CTGATCCCACCACGAAAACT
GNG3	Forward	CCGGTGAACAGCACTATGAG
	Reverse	TGCCTTGGACACCTTTATCC
ITIH3	Forward	AAGGAAGTTGCCAAGAAGCA
	Reverse	GTGATGAAAGAGGCCTCAGC

Table S3：Human specific siRNA.

Gene	Sequence of siRNA（5’ to 3’）
siGNG3-1	GTGTCGGATAAAGGTGTCCAA
siGNG3-2	GCTTAAGATTGAAGCCAGCTT
siPRXL2A-1	GAGACAAAGTAAACCTACTTT
siPRXL2A-2	CTTTCAAAGCAAAGGAGCTAT
